# Challenging existing Raven's norms based on children attending government schools in India

**DOI:** 10.1007/s41809-026-00207-y

**Published:** 2026-06-15

**Authors:** Margreet Vogelzang, Mandy Wigdorowitz, Ianthi M. Tsimpli

**Affiliations:** 1 Department of Theoretical and Applied Linguistics, University of Cambridge, 9 West Road, Cambridge, CB3 9DA UK; 2 School of Psychology, Newcastle University, Dame Margaret Barbour Building, Wallace St, Richardson Rd, Newcastle Upon Tyne, NE2 4DR UK; 3 Department of Modern Languages and Classics, University of Alabama, 200-C B.B. Comer Hall, Tuscaloosa, AL 35487 USA; 4 Department of Psychology, University of Johannesburg, Umthombo Building, 1 Jan Smuts Avenue, Johannesburg, 2001 South Africa

**Keywords:** Raven's coloured progressive matrices, Intelligence test, Visuo-spatial reasoning, Norm-referenced scores, India, Government schools

## Abstract

Intelligence measures, such as the Raven's Coloured Progressive Matrices (CPM), rely on norm-referenced scores to interpret individual performance and guide educational or clinical interventions. However, when norm comparisons result in a severely skewed distribution, the validity of the norm sample must be scrutinized rather than accepting implausible interpretations. In this study, we challenge existing norms and improve the suitability of the CPM for Indian children attending government schools by developing new norms that better reflect their socio-demographic and educational contexts. Our sample includes 1,752 children in Delhi, Hyderabad, and Patna from two large-scale projects. Using existing CPM norms for India, we found that 78.5% of our sample scored in the bottom 10th percentile with an IQ score of 80 or lower, highlighting a heavily skewed distribution. Upon reviewing the existing norm sample, we identified several limitations: its size was five times smaller than our sample, the sample’s linguistic background was underspecified, and it predominantly represented children from higher socio-economic backgrounds attending private schools. This norm sample fails to represent the majority of Indian pupils, particularly those in government schools, who constitute approximately two-thirds of the pupil population. In response, we developed alternative norms across four age categories (8, 9, 10, 11) that better align with the demographic realities of this group. These norms demonstrate an expected distribution of scores and provide a better benchmark for evaluating intelligence, specifically visuo-spatial reasoning, of Indian children attending government schools. We encourage researchers, educators and clinicians to use these norms as appropriate.

## Introduction

Having norms for measures of intelligence (*g*) and other psychological constructs serves as a reliable and valid way to fairly compare, position, and identify potentially vulnerable individuals against a representative sample. Norm-referenced scores provide a standard against which individual test scores can be interpreted, making it possible to determine how an individual's score fares against that of a similar group and, in turn, provides a marker of performance that can be used for educational or clinical intervention. Comparison of a sample against the respective norm group should follow a Gaussian distribution whereby the majority of the sample scores within an average range and a smaller proportion either excels or scores poorly. Norm-referenced scores capture this normal variation and can be used to appropriately classify the performance of individuals against which the norm sample claims to generalize. But what happens in cases where reference to norm scores disproportionately renders a large proportion of children as poor or vulnerable performers? Does such an interpretation suggest that a large group of children which are compared against the norms have intellectual challenges or risks, or are the norms themselves perhaps unrepresentative of the population they claim to represent? In this paper, we take the latter stance by (1) providing evidence showing that a group of Indian children attending government schools fare markedly poorer than expected against the Indian Raven’s norm-referenced scores, and in response, (2) creating more representative Raven's norms for this population of Indian children. We aim to improve the suitability of Raven's Coloured Progressive Matrices for Indian government primary school pupils by establishing norms that are more reflective of their demographic, geographic, and educational backgrounds, which encompass variables identified to affect norm-referenced scores in children (Reynolds & Mason, 2009). Below we provide an overview of what the Raven’s Progressive Matrices measure, including how and for whom they have been used. We then explore the cultural factors that influence performance and examine the role of socio-demographic indicators. Additionally, we discuss the broader educational and socio-economic context for children in India, particularly those attending government schools, and highlight the issues with the current CPM norm-referenced scores.

### Cultural and socio-economic status influences on Raven's performance

The Raven’s Progressive Matrices (RPM) are measures of fluid intelligence, designed to capture (non-verbal) visuo-spatial reasoning without relying on test-takers’ prior knowledge, education, or experience. RPM does not rely on linguistic ability but rather uses visual material that requires the selection of a correct image from an array of options that adheres to a pattern. There are three standardized versions of the RPM, namely (1) the Standard Progressive Matrices (SPM) which is suitable for the general population for individuals aged 6 and over who have average intelligence, (2) the Advanced Progressive Matrices (APM) which is a more difficult version of the test and is suitable for people with above-average intelligence aged 12 and older, and (3) the Coloured Progressive Matrices (CPM) which is designed for children between the ages of 4 to 11 and can also be used to assess visuo-spatial reasoning of elderly individuals and people with learning difficulties (Raven, 2003). Each version has been designed as fit-for-purpose and appropriate for particular populations, ensuring that non-verbal intelligence can be measured for a wide range of age groups and ability levels. Scores positively increase with age (up to a certain point; Smirni, 2020), and it has been observed that people are obtaining higher intelligence test scores over time with an average increase of three points per decade (known as the ‘Flynn Effect’, Flynn, 2012).

Since its development by John C. Raven in 1936 (Raven, 1936, 1941), all versions of the RPM have been widely employed and normed across various global contexts for experimental, educational and clinical means to ensure its ecological validity (Brouwers et al., 2009; Cotton et al., 2005; Raven, 2000). Because of its non-verbal design and easy implementation, the RPM is thought to be applicable cross-culturally for children and adults and not confounded by cultural or national differences such as level of education, socio-economic status, and other exogenous factors (e.g., Rushton et al., 2004; Valencia, 1984). However, it has been argued that the RPM cannot be culturally neutral because intelligence itself and its assessments are intrinsically cultural constructs (Frijda & Jahoda, 1966). Such cultural loading – the extent to which a test or test item reflects the values, language, information, and experiences of a specific cultural context – inherently plays a role in performance (Jensen, 1974). In fact, some studies suggest that visuo-spatial tests, like the RPM, are influenced by culture to a greater extent than verbal tests (Ardila & Moreno, 2001; Rosselli & Ardila, 2003) and are significantly affected by training (Jaeggi et al., 2008), repeated testing (Ombrédane et al., 1956; Wing, 1980) and bilingualism (Bialystok & Shapero, 2005; Tsimpli et al., 2020a). To address performance disparities and such method biases, a distinction has been drawn between the construct of *intelligence* itself and *intelligence test scores*, whereby competence can be seen as separate from performance (Van de Vijver & Leung, 1997).

In response to ensuring culturally responsive testing practices, there is ample research investigating the psychometric soundness and applicability of the RPM across various populations and cultural contexts. However, its development, adaptation, and the majority of norming, educational, and clinical studies come from W.E.I.R.D. (Western, Educated, Industrialised, Rich and Democratic, Henrich et al., 2010) samples and contexts, predominantly in the Global North (Brouwers et al., 2009; Raven & Raven, 2000). In cases where the RPM is evaluated in countries or cultures that are not considered W.E.I.R.D., there are notable concerns questioning its cultural neutrality, fairness, and appropriateness (for a review, see Gonthier, 2022). This is largely because test-takers regularly perform suboptimally in comparison to norm reference groups or their W.E.I.R.D. test-taker counterparts. For example, in many African countries, RPM scores are considerably lower than expected, with some even falling below 1 standard deviation of the mean (see for example, Ghana: Anum, 2014; Kenya: Costenbader & Ngari, 2001; Libya: Al-Shahomee & Lynn, 2010; South Africa: Knoetze et al., 2005). Furthermore, some properties of the RPM do not hold up to psychometric standards in non-W.E.I.R.D. contexts, as illustrated in a review of the RPM in African samples (Wicherts et al., 2010). Cultural confounds have also been found to contribute to different scores for different ethnic and gender groups which have led to unsubstantiated and damaging conclusions about gender/race and intelligence in the past (see Lynn & Vanhanen, 2006; Raven & Raven, 2000; Wicherts et al., 2010). However, gender differences are not always observed (Kazem et al., 2009; Raven, 2012).

In no way is the poorer performance of non-W.E.I.R.D. samples on the RPM indicative of innate difficulties in visuo-spatial reasoning; rather, it reflects biases and the cultural appropriateness of the test itself. Non-verbal intelligence tests rely on the manipulation of some type of visual material (e.g., puzzles), but if such material or its perceptual manipulations differ, are limited, or absent in home, pre-school, or school contexts, then it is no surprise that test-takers would be disadvantaged if they are required to transfer specific skills which have not been honed. Simply engaging with the RPM requires prior experience with visuo-spatial processes, gestalt reasoning and an understanding of test taking in general. From knowing the names and representations of varying geometric shapes to recognizing numerical cues and more complex symbolic representations, a myriad of reference points are needed to decipher Raven’s items (Ardila & Moreno, 2001; Pontius, 1995). In addition, there are possible language barriers: although the test itself is considered non-verbal, language is essential for relaying the test instructions. Collectively, these processes rely on previous experience and engagement with a specific mode of representation such that greater experience translates to greater ease of item manipulation. Being at least critical and cautious about a blanket assumption of cultural fairness in intelligence testing is therefore warranted when evaluating scores either in their raw form or against a norm.

While re-standardization and validation of assessments in local populations can reduce cultural biases and result in the development of local norms, such measures do not completely remove the effects of exogenous factors on test scores. One of the most significant predictors of RPM performance are socio-demographic indicators, such as socio-economic status (SES). SES, while measured in different ways, provides an estimate of social affluence and serves as a proxy for one's access to resources and opportunities within a society. SES has been widely studied and found to be positively related to language development, educational attainment, health outcomes and many other population-level variables (e.g., Brito & Noble, 2014; Broer et al., 2019). Importantly, SES has been linked to fluid intelligence performance, with individuals in lower SES groups underperforming those in higher SES groups (for a meta-analysis, see Peng et al., 2019). In a study investigating the impact of SES on fluid intelligence in Ghanaian children (6–12 years old), Anum (2022) found a disparity in performance on the CMP where children attending private schools (a proxy for higher SES) scored higher than those attending public schools (a proxy for lower SES). These findings demonstrate the strong influence that SES can have on the RPM performance in children, particularly in developing countries, and raises an important question about whether single and blanket norm-referenced scores can adequately capture visuo-spatial reasoning of children where disparities in SES are pronounced and evident in terms of educational access and equity. Such a distinction also exists in India, where schooling quality is largely determined by economic resources (Narwana et al., 2026,1). Culturally appropriate norms for diverse groups of people in the context of India are therefore needed. In the next section, we review the educational system in India and discuss the development and application of the Indian CPM along with its limitations.

### Education and Raven’s in India: insights and issues

India is the world’s most populous country with an estimated population of nearly 1.5 billion people, of which approximately 25% (370 million) are children between the ages of 0–14 (The World Bank Group, 2025). Roughly two-thirds of school-going children in India attend government schools, with the remaining third attending private schools (Government of India, 2021–22). India is a highly multilingual and diverse country and is home to over 424 indigenous languages, of which 22 are considered ‘scheduled’ and used for official purposes (e.g., Telugu, Kannada) with English serving as a link language alongside Hindi (Census of India, 2011; Eberhard et al., 2024). It also has one of the highest linguistic diversity indexes of 0.914 as reported by UNESCO (2009; range 0–1). About 27 languages, including English, are used as mediums of instruction across government schools depending on the location and local language(s) of the region (Eberhard et al., 2024; Mohanty, 2006). Private education, on the other hand, prioritizes English as the primary medium of instruction because of its esteemed perception (a side-effect of colonial history) and association with economic and social mobility.^2^

A standardized version of the Raven’s CPM (Ravens Educational CPM/CVS(India)) has been normed by Pearson Clinical India for Indian children (Raven, 2012; see Table 1 for an overview of the norm sample). Importantly, all children in the norm sample attended private schools, so children attending government schools were not included or represented in the norm population. In addition, 94.1% of the children’s parents had obtained a diploma/graduate degree or higher, suggesting that the norm population predominantly represented children from higher socio-economic standing (Ghosh & Dey, 2020). Finally, the linguistic background of the norm sample was underspecified and represents “the English speaking Indian population of children, attending English medium schools” (Raven, 2012, p. 36). This description is rather vague and can be interpreted in many ways, including first language (L1) English speakers (who comprise less than 0.02% of the population; Census of India, 2011), second language (L2) English speakers (who comprise about 30% of the population and who may differ greatly in proficiency; Census of India, 2011), additional language English speakers (of a third, fourth etc. language), and speakers who use the Indian English variety (Sharma et al., 2017).

**Table 1:** Comparison of the variables characterizing the sample used to create the Pearson India norms and our sample

Variables	Pearson norm study	Current study
Sample size	338	1,752
Age	4–11;11	8–11;11
Gender	164 girls (48.5%), 174 boys (51.5%)	982 girls (56.1%), 770 boys (43.9%)
Type of school	Private	Government
Socio-economic status	High; 94.1% of parents had a diploma/graduate degree or above	Low; all children attended (free) government schools. Parents' occupations also suggest SES is not high
Regions	Bhopal, Delhi, Lucknow, Patna, Bengaluru, Chennai, Hyderabad, Kolkata, Ahmedabad, Mumbai, Guwahati	Delhi, Hyderabad, Patna
Official Medium of Instruction school	English	Regional languages (68.6%), English (21.6%), English and a regional language (9.8%)
Languages spoken	English speaking Indian population (other languages not specified)	Children spoke a variety of mother tongues, including Hindi (*n* = 749 monolingual speakers, *n* = 292 multilingual speakers with Hindi as their first language), Telugu (*n* = 185 monolingual speakers, *n* = 277 multilingual speakers with Telugu as their first language), Bhojpuri (*n* = 34), Lambadi (*n* = 21), Kannada (*n* = 13), and Rajasthani (*n* = 10). Only 3 children reported speaking English as their first language
Language of instruction of test	Not specified, but likely English (“Children were assessed only if they could read, write, and speak English”; Raven, 2012, p. 39)	Children instructed in the language they were most comfortable in, insofar as this was possible ^a^

^a^Non-English instructions, translated by the Research Assistants, were predominantly in Hindi (Delhi and Patna) and Tegulu (Hyderabad), and were limited by the languages the Research Assistants spoke. Since instructions for this test are relatively simple, we are confident that the translations did not affect the children’s understanding or performance on the test

Existing CPM India norms are therefore unrepresentative of a large proportion of Indian children, including those who attend government schools and who come from lower SES backgrounds. This was already argued by Tsimpli et al. (2020a), who explicitly indicated that the standard scores from Raven (2012) may not be representative of their sample of low-SES children and therefore used raw (non-standardized) performance scores for their analyses.

Following the recommendations from the International Test Commission (2015), “a test is obsolete when its underlying theory, item content, norms, or technical adequacy no longer meet the needs for its intended purpose, professional standards, or when its continued use would lead to inappropriate or inaccurate decisions or diagnoses” (p. 15). While we do not wish to discard the existing CPM test and Indian norms altogether, it is clear that they are not fit-for-purpose for Indian children attending government schools and are in fact obsolete as a reference for the majority of Indian children more generally. Nevertheless, the test itself still seems meaningful, for example because it showed a correlation of .27 with a different cognitive task, namely the n-back task, in a population of Indian children attending government schools (Tsimpli et al., 2020a). Therefore, we will combine existing data from Tsimpli et al. (2020a) and a follow-up study which both used the CPM with Indian children attending government schools to generate alternative CPM norms that more appropriately reflect the demographic background of this population.

## Methodology

For the analysis and interpretation of our data, we followed the GRoNC (Guidelines for Reporting on Norm-Referenced and Criterion-Referenced Scores) procedure proposed by Timmerman et al. (2025).

### Sample

For this study, data from two sources were combined. A large part of the dataset (*n* = 1,581) comes from the *Multilingualism and Multiliteracy in India (MultiLiLa)* project (2016–2020; Tsimpli et al., 2019, 2020a). Additional data from children in Hyderabad (*n* = 171) are part of the follow-up project *Supporting the development of Indian primary school children's reading comprehension skills: A SCaffolding-based Intervention* (2021–2022; Vogelzang et al., 2024a; henceforth referred to as the *ASCI* project)^3^. Our final sample consists of 1,752 children across four age categories (age 8, 9, 10, and 11; 982 girls, 770 boys, see Table 2 for an overview), which is considered a ‘good’ sample size for the development of norm-referenced scores overall (Evers et al., 2009) and within each age group (Lenhard et al., 2019). All participants attended year 4 or 5 in government primary schools in India. The participating primary schools were located in Delhi, Hyderabad (highly urban), and Patna (more rural). These three cities were selected for the projects to reflect, at least to some extent, the geographical and cultural variation present in a country as large and diverse as India. Inclusion of schools in the projects were based on geographical region and type of school/SES. Concerning the latter, only government schools were recruited, which are free to attend and almost always have pupils from low SES backgrounds (Narwana et al., 2026). This contrasts with the sample used in the original Raven's norms for India, which was based on private schools which are paid and typically have pupils from medium to high SES backgrounds (see Table 1 for a comparison between the two samples).

**Table 2:** Overview of the number and percentage of children in our sample per age group and city

		Age 8	Age 9	Age 10	Age 11	Total
Delhi	*n*	109	224	17	3	353
	Girls	60 (55%)	109 (49%)	9 (53%)	0 (0%)	
	Boys	49 (45%)	115 (51%)	8 (47%)	3 (100%)	
Hyderabad	*n*	84	185	202	130	601
	Girls	45 (54%)	112 (61%)	114 (56%)	66 (51%)	
	Boys	39 (46%)	73 (39%)	88 (44%)	64 (49%)	
Patna	*n*	82	261	309	146	798
	Girls	45 (55%)	160 (61%)	180 (58%)	82 (56%)	
	Boys	37 (45%)	101 (39%)	129 (42%)	64 (44%)	
Total		275	670	528	279	1,752

The relatively low SES backgrounds of the children in our sample is confirmed when looking at their parents’ occupations. From the children for which information on this was accessible to us (*n* = 872, 49.8%), for the overwhelming majority either one or both of the parents were laborers, with occupations such as cab or auto driver (*n* = 124), some type of seller/vendor (*n* = 103), mason or construction worker (*n* = 86), painter (*n* = 57), or general (day) laborer (*n* = 46) for the fathers and maid or house help (*n* = 137) or seamstress (*n* = 22) for the mothers. These occupations formed the largest categories, but this is not an exhaustive list. Data from the *ASCI* project additionally show that 15% of children indicated that their father could not read or write and 27% indicated that this was true of their mother. In addition, 20% of children indicated that their father had not been to school (note that this does not refer to a diploma/degree, as in the existing Raven's norms, but to primary/secondary school), and 24% indicated this for their mother. Given all these factors, we are confident that these children represent a sample from relatively low to very low SES backgrounds.

### Procedure

The Raven's CPM test (Raven et al., 2008) consists of 36 items (matrices). In each item children were presented with a pattern from which one piece is missing (reminiscent of a puzzle). They are asked to pick one of six possible 'puzzle' pieces (answer alternatives) to complete the pattern – only one answer is correct, the other five are incorrect. The 36 matrices are divided equally into three sets (A, AB, and B) and items become progressively more difficult. Each correct answer gains the child 1 point, with the maximum possible score being 36.

The matrices and answer alternatives were presented on a laptop, and children could indicate their choice by saying the number of the answer alternative or by pointing to it. Besides the explanation of the test, it is thus, or at least it has the potential to be, a non-verbal test. The tests were administered by research assistants from India who were proficient in local languages as well as English. The children were tested individually in a separate room or courtyard in their school. The test explanation was provided in the language they were most comfortable in, insofar as that was possible given the many languages spoken by the children. Demographic and language background information was obtained through an interview with the children, also in the language they were most comfortable with insofar as it was possible.^4^

### Ethical considerations

Ethical consent was collected as part of the two projects that the samples were taken from. Consent was given by the child (orally), as well as by the head teacher or principal of the participating schools. Many of the parents of lower-SES children in India are semi-literate or illiterate and their communication with the school is scarce. Parental consent was therefore ensured through the school principal and the children's teachers. The principal approached parents (mostly by phone) to ask them for consent. The studies were conducted in accordance with the Declaration of Helsinki, the ESRC’s Framework for Research Ethics (ESRC, 2010), and the guidelines of the Indian Council for Medical Research (ICMR, 2006). The protocol of the *MultiLiLa* project was approved by the Ethics Committees of the University of Cambridge (RG83665), the Jawaharlal Nehru University, and the National Institute of Mental Health and Neurosciences. The protocol of the *ASCI* project was approved by the Ethics Committee of the University of Cambridge (2019–20/65). The project additionally obtained permission to approach schools from the State Minister of Education in Telangana (the state in which Hyderabad is located).

### Analysis

The data from the two projects was prefiltered based on (1) availability of the Raven's CPM, (2) the age range of the children (i.e., children under 8 [*n* = 14 of age 7] were excluded because of insufficient data, children over 11 were excluded because they were overaged for their grade level [*n* = 159]), and (3) the type of school (the *ASCI* project included three low-cost private schools which were not included in the current sample). The data contained total raw scores but no by-item information.

The new norms and all plots presented in this paper were created in the R software (version 4.4.1, R Core Team, 2019). We followed the tutorial by Timmerman et al. (2021) and used the package *gamlss* (Rigby & Stasinopoulos, 2005) to calculate the new norms. We used a Generalized Additive Model for Location, Scale, and Shape (GAMLSS) with a Box-Cox power exponential distribution, and computed the norm-referenced scores for the scale based on the estimated model. Our model used age as a regressor, which allows the new norms to increase logically with age. Since our dataset only contained age in years (i.e., age 8, 9, 10, and 11) without month-level data, we will report the new norms in terms of age in years as well, however, within-year developmental variation is also estimated by the model (and can be obtained from the OSF script). The norm calculations return continuous standard scores following a normal distribution (from 55 to 145 with a mean of 100 and standard deviation of 15), but are presented in the manuscript more concisely (as in the Pearson's versions of Raven's norms) in increments/bins of 5. In doing so, numbers were rounded: for example, scores for 98 to 102 became bin 100. All plots were created with base-R. For the plots of the standard scores, standardized scores of < 60 were coded as 55 and standardized scores of > 140 were coded as 145.

We then calculated the means, ranges, and standard deviations of the Raven's scores for the sample. Skewness and Kurtosis values were calculated with the *moments* package (Komsta & Novomestky, 2022). As a rule of thumb, if the skewness is between -0.5 and 0.5, the data are fairly symmetrical. If the Skewness is between -1 and -0.5 or between 0.5 and 1, the data are moderately skewed. If the skewness is less than -1 or greater than 1, the data are highly skewed (Bulmer, 1979). For Kurtosis, the value reflecting an optimal normal distribution is 3. The greater the Kurtosis, the higher the peak.

We estimated the reliability of the Raven's CPM test for our sample of participants by examining the test–retest correlation of a subset of 629 children using Spearman's correlation. We additionally did a random split comparison: we split the dataset of children for each age category in half and compared whether their means differed significantly with an independent samples (unpaired) *t*-test.

To verify whether performance improved with age, we ran a linear regression with raw score as the dependent variable and age as the independent variable. Finally, we ran independent samples (unpaired) *t*-tests to compare the performance of girls and boys in each age group.

The full dataset and analysis code for this paper can be found on the OSF: https://osf.io/4tm3u/overview?view_only=434e59262a654fc4b25d2c144d0da179.

## Results

We first discuss the current dataset and why the existing norms do not apply to it. We then present alternative norms for children attending government schools in India. Finally, we present some measures of reliability and construct validity of the Raven's CPM for this sample of children.

### The current dataset and existing norms

**Fig. 1:**
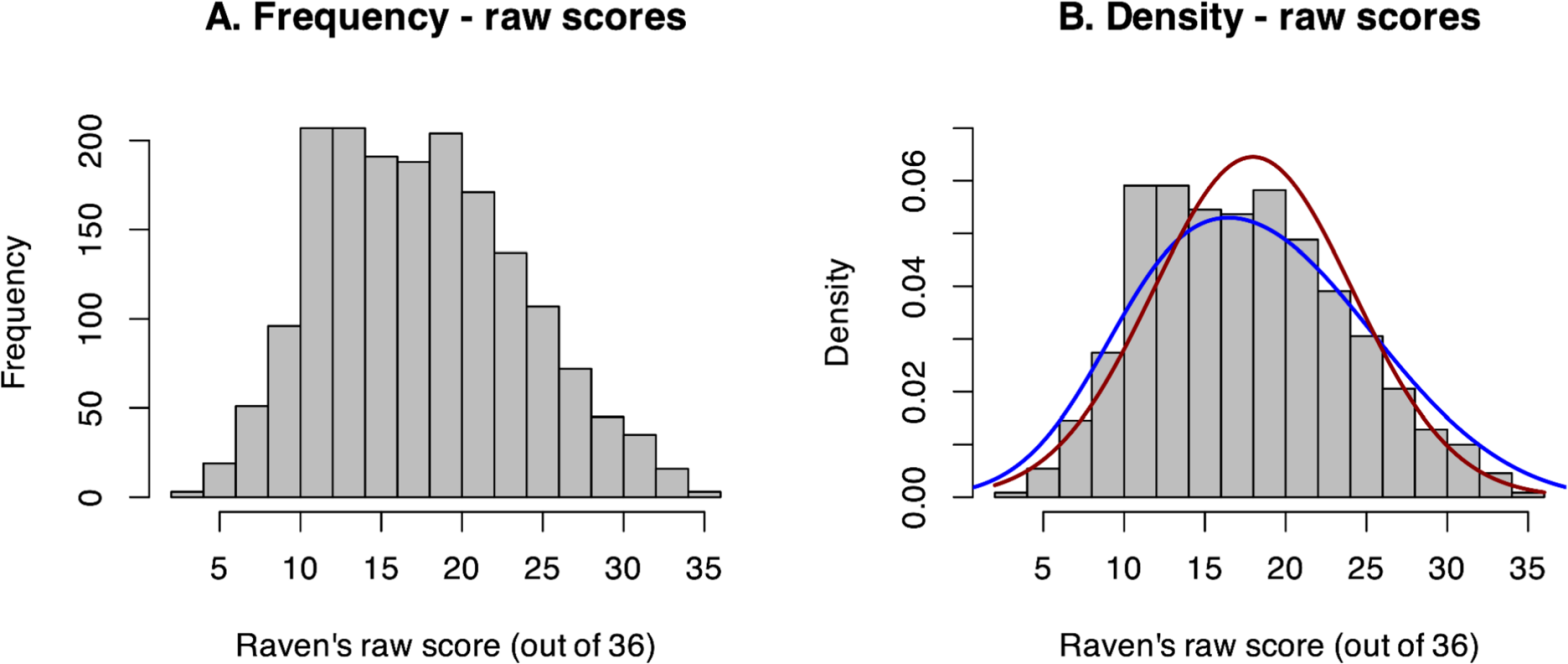
Histogram (A) and density plot (B) of the children's raw scores on the Raven's CPM test. The density plot includes estimates of the normality of the distribution, with the empirical (observed) distribution shown in blue and the idealized normal (Gaussian) distribution based on the mean and standard deviation of the empirical data in red

Some core descriptives of the current dataset, including children’s raw scores on the CPM, per age category are presented in Table 3. The mean raw scores on the test tend to increase with age, as expected (Smirni, 2020). The Skewness and Kurtosis of the raw scores are within the acceptable range of normality. The raw scores indeed visually resemble a normal distribution (see Fig. 1).

**Table 3:** Descriptive statistics of our sample (*N* = 1,752) in the Raven's CPM test, including measures of Skewness and Kurtosis

		**Raw scores**					**Standard scores**	
Age	***n***	**Mean**	**Range**	**SD**	**Skewness**	**Kurtosis**	**Skewness**	**Kurtosis**
8	275	17.19	5–35	5.87	.47	2.98	.68	2.87
9	670	17.89	2–35	6.06	.32	2.59	.76	2.86
10	528	17.82	4–35	6.07	.31	2.57	1.09	3.75
11	279	19.28	5–34	6.80	.10	2.18	.92	2.84

**Fig. 2:**
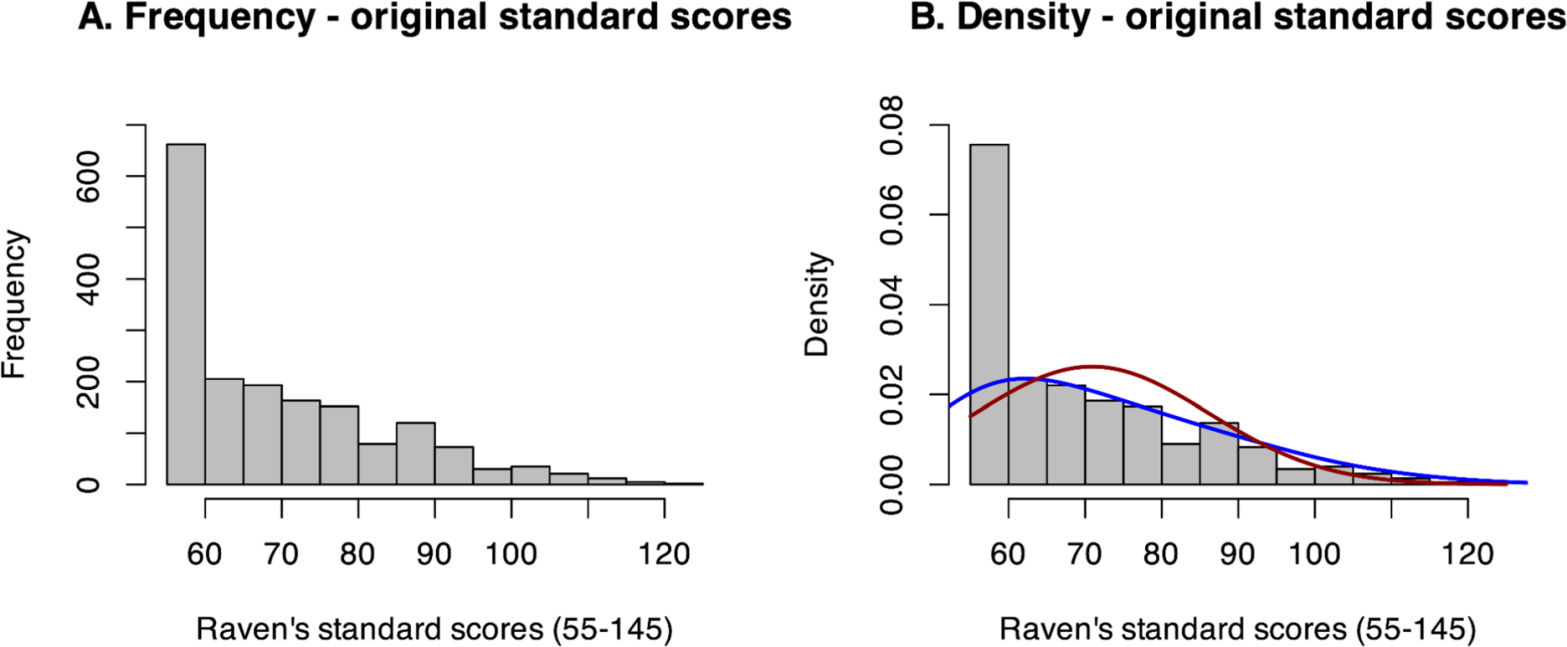
Histogram (A) and density plot (B) of the children's standard scores for all ages together according to the existing Raven's CPM norms for Indian children. The density plot includes estimates of the normality of the distribution, with the empirical (observed) distribution shown in blue and the idealized normal (Gaussian) distribution based on the mean and standard deviation of the empirical data in red

If the existing norms would be appropriate for this sample of children, the normed standard scores should also resemble a normal distribution. However, this is not the case (see Fig. 2). In fact, 1,375 of the children (78.5%) scored in the bottom 10th percentile, making the distribution heavily skewed and flagging the majority of children as potentially having intellectual challenges, with a standardized (IQ) score of 80 or lower (e.g., Wieland & Zitman, 2016). Measures of Skewness (Table 3) confirm that the standardized scores are moderately to highly right-skewed. Thus, the existing norms are too stringent for this sample of children and lead to potentially meaningful distinctions between children's scores being lost when interpreted by reference to normed data. For example, children of age 11 who score 16 or below are all assigned the standard score < 60, with a percentile rank of 0.1, even though a child scoring 16/36 objectively performed better than a child scoring 10/36.

### New norms for children attending government schools in India

**Fig. 3:**
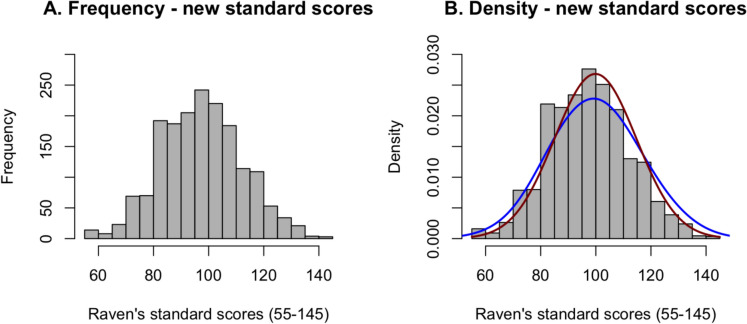
Histogram (A) and density plot (B) of the children's standard scores for all ages together after using the new Raven's CPM norms for Indian children. The density plot includes estimates of the normality of the distribution, with the empirical (observed) distribution shown in blue and the idealized normal (Gaussian) distribution based on the mean and standard deviation of the empirical data in red

**Fig. 4:**
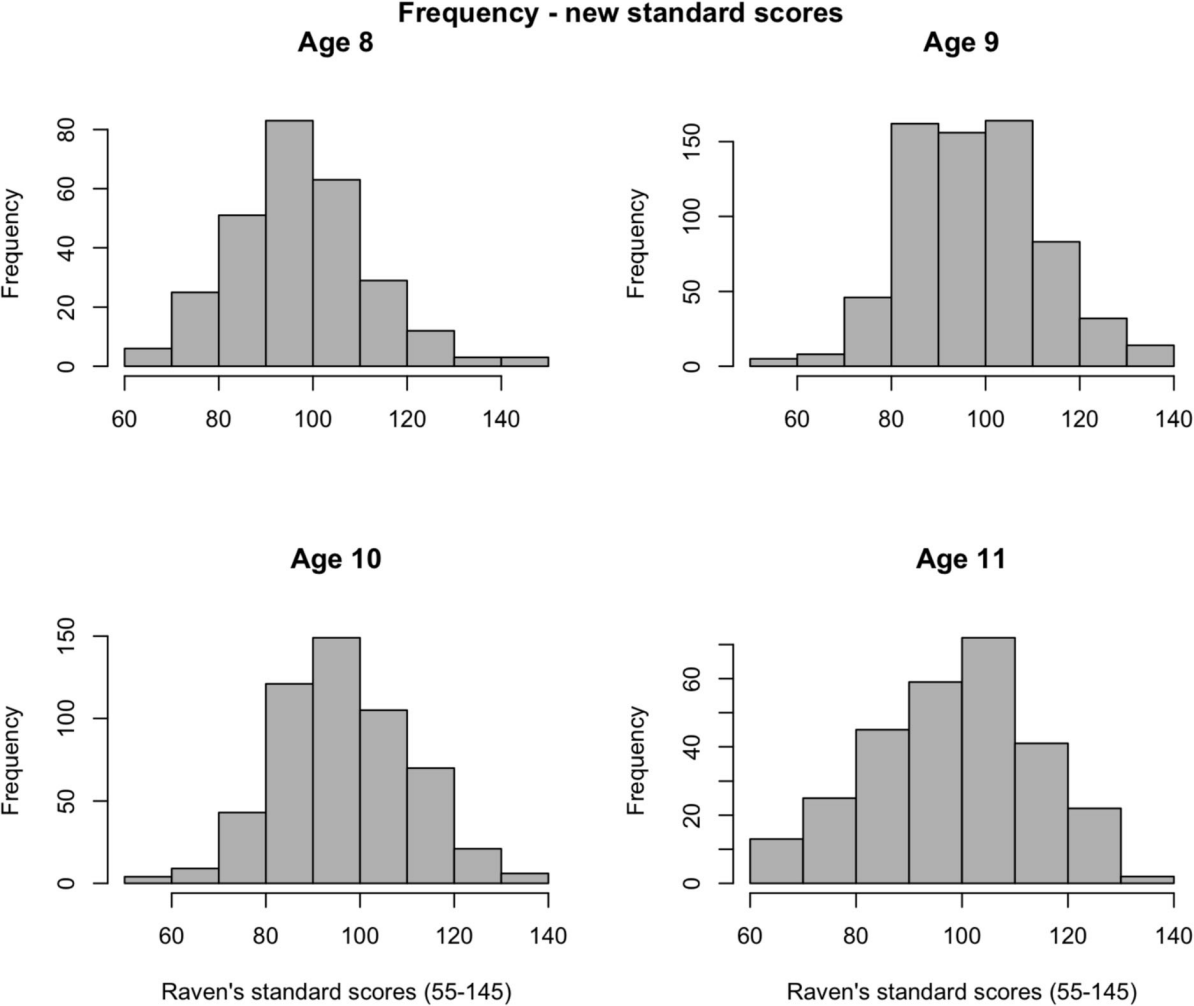
Histograms of the children's standard scores at each age (8, 9, 10, 11) after using the new Raven's CPM norms for Indian children

Based on the children’s raw scores, and the assumption that scores should be normally distributed, new norm-referened scores were calculated for these 8-to-11-year-old children (see Table 4). When using these new norms to calculate standard scores, the scores approach a normal distribution (compare Figs.2, 3). For completeness, Fig. 4 shows the distributions of the standard scores for the four different age groups.

**Table 4:** New Raven’s CPM norms table for 8-to-11-year-old children attending government schools in India

Standard score	Percentile rank	Age 8	Age 9	Age 10	Age 11
< 60	**0.1**	0–4	0–4	0–4	0–4
60	**0.4**	5	5	5	5
65	**1**	6	6	6	6
70	**2.3**	7	7	7	7–8
75	**5**	8–9	8–9	8–9	9
80	**9**	10	10	10	10–11
85	**16**	11–12	11–12	11–12	12
90	**25**	13	13–14	13–14	13–14
95	**37**	14–15	15–16	15–16	15–17
100	**50**	16–18	17–18	17–19	18–19
105	**63**	19–20	19–20	20–21	20–22
110	**75**	21–22	21–23	22–23	23–24
115	**84**	23–24	24–25	24–25	25–26
120	**91**	25–26	26–27	26–28	27–29
125	**95**	27–28	28–29	29–30	30–31
130	**97.7**	29–30	30–31	31–32	32–33
135	**99**	31–32	32–33	33–34	34–35
140	**99.6**	33	34–35	35–36	36
> 140	**99.9**	34–36	36	-	-

We additionally examined the reliability and construct validity of the Raven's CPM in our sample of children. Note that these investigations were performed on the children’s raw scores (out of 36) and are thus not a validation of the norms, but rather of the Raven's CPM test itself.

### Reliability

We analyzed the external reliability of the Raven's CPM in two ways. The first was through a test–retest paradigm. The Raven's CPM test was re-administered to a subsample of 629 children (338 from Delhi, 291 from Hyderabad; 334 girls, 295 boys) after approximately one year. The correlation coefficient between the two administrations was .52 (CI = [.46—.57]). This is a little lower than was found for other studies (e.g., Kazem et al., 2009), but the time period in between the two tests was much larger in the current study. However, it is also lower than the previously reported one-year test–retest reliability (.71, Court & Raven, 1995, as reported in Kazlauskaite & Lynn, 2002), and more in line with previously reported two-year test–retest reliability (of .499 in Lithuanian children, Kazlauskaite & Lynn, 2002). Notably, children scored around 3 points higher on the retest (*M* = 21.40; Median = 21) than on the first test (*M* = 18.45; Median = 18).

For an investigation of robustness across subsamples, we implemented a random split comparison. That is, we split the dataset of children for each age category in half randomly and compared whether their means differed significantly. The results indicate that this was not the case for any of the age groups (see Table 5).

**Table 5:** T-test results comparing random subgroups on Raven’s CPM in the four different age categories

Age	*n*	1st half Mean (*SD*)	2nd half Mean (*SD*)	*t*	*df*	*p*
8	275	17.04 (5.51)	17.33 (6.22)	-.41	270	.683
9	670	17.71 (5.83)	18.06 (6.28)	-.75	664	.456
10	528	17.76 (5.83)	17.89 (6.32)	-.25	523	.802
11	279	18.92 (6.29)	19.63 (7.28)	-.87	272	.385

### Construct validity

We assessed the construct validity of the Raven's CPM for our sample by assessing whether performance improves with age and whether performance differs between boys and girls. A linear regression confirmed that raw scores significantly increased with age (*ß* = .56; *t* = 3.55; *p* < .001) as expected.

We then assessed the influence of gender in each age category. T-tests showed no difference in performance between girls and boys at age 8, but differences in performance between girls and boys at age 9, 10, and 11 were found, as well as when all ages were collapsed, with boys outperforming girls (see Table 6).

**Table 6:** Comparison between performance on the Raven's CPM by boys and girls across the four age categories

Age	Girls: Mean (SD)	Boys: Mean (SD)	*t*	*df*	*p*
8	16.95 (5.75)	17.47 (6.01)	-.73	260	.468
9	17.09 (5.86)	18.93 (6.16)	-3.92	603	< .001 ***
10	17.09 (5.95)	18.82 (6.10)	-3.26	476	.001 **
11	18.48 (6.35)	20.18 (7.19)	-2.08	261	.039 *
all	17.28 (5.96)	18.87 (6.35)	-5.37	1600	< .001 ***

^*^ *p* < 05; ** *p* < .01; *** *p* < .001

## Discussion

This study challenged the existing Raven’s CPM norms for India (Raven, 2012) based on a sample of 8-to-11-year-old Indian children who attend government primary schools. We argued that the existing norms were developed based on children with higher SES attending private schools, whereas at least two-thirds of school-going children in India attend government schools and are from lower socio-economic backgrounds (Government of India, 2021–22). According to the existing norms, the vast majority of children in our sample substantially underperformed compared to the sample on which the existing norms were based (cf. Al-Shahomee & Lynn, 2010; Anum, 2014; Costenbader & Ngari, 2001; Knoetze et al., 2005). This interpretation is harmful and does not adequately identify children who are in fact intellectually at risk and who are performing in line with or better than their peers. We presented new norms (Table 4), which are more appropriate for our sample and potentially for other Indian children between 8–11 years old who attend government primary schools as well. We strongly encourage researchers who use CPM standardized scores to think about which norms are most appropriate for their sample and for educators and clinicians to use the norms most appropriate on a case-by-case basis.

Furthermore, we presented evidence that the Raven’s CPM test in our population is reasonably reliable based on test–retest and split halves paradigms. We are thus confident that the test can be a useful tool for testing Indian children. Importantly, although we provided an overview of several issues with the Raven’s tests for diverse populations in the Introduction, we are not advocating to abandon it altogether in non-W.E.I.R.D contexts given that it still provides some useful information about visuo-spatial reasoning. Rather, we are (1) providing alternative norms that more appropriately reflect a large proportion of Indian children, and (2) cautioning researchers, educators, and clinicians to carefully consider what the outcomes of a Raven’s test in their participants may signify, ensuring they account for the broader (sociocultural) context and possible influences. Importantly, we support the view that tasks such as the RPM can give insights into *intelligence test scores*, which are separate from *intelligence* itself (Van de Vijver & Leung, 1997). However, to be more specific, and to avoid any potential misunderstanding, it is more accurate to regard the Raven’s CPM as assessing visuo-spatial reasoning in children by means of puzzle manipulation rather than intelligence (*g*) more broadly (Gonthier, 2022).

Regarding construct validity, we found differences between boys and girls. This result is in contrast with previous research which has generally found invariance of performance across genders on Raven’s CPM (e.g., Kazem et al., 2009). In fact, the sample of children used in the original norms for India showed a non-significant difference in performance between boys and girls (collapsed over age groups, *p* = .44; Raven, 2012). However, the result in this study is in line with Hervé et al. (2022), who found a similar gender gap in cognitive (and non-cognitive) outcomes, including Raven’s, in Indian adolescents. They suggest that this is due to “an institutionalized gender bias in education against girls in India” (p. 85). Although we cannot know the cause of this for certain, we can speculate about why boys outperformed girls in the sample presented in the current study. Specifically, this might be a result of the culture within lower socio-economic groups in India, with education being more challenging for girls in terms of regular school attendance and continuity. However, we have no direct evidence for this in the current sample, and there were more girls (*n* = 982) than boys (*n* = 770) attending the schools from which our data were collected. Girls in India, especially from lower SES backgrounds, have been known to outperform boys in language subjects (Natta et al., 2017; Shenoy et al., 2023; UNICEF, 2012; Vogelzang et al., 2024b). Interestingly, in previous research using a subsample of our study (Tsimpli et al., 2020a), gender was not found to influence performance on an n-back cognitive task or Raven's CPM. So, the observed effect of gender needs further investigation to be substantiated.

Note that the new norms presented in this paper are not all-encompassing either. Specifically, they may lead to potential skewness in the opposite direction when used on a sample of higher-SES children and/or those attending private schools or residing in different regions across India, causing researchers, educators, or clinicians to miss children who are indeed in need of intervention. A truly representative norm would be one that includes all children across socio-economic and geographic strata in India, but given the limitations with the Pearson norms, and the vastness of India’s populace and geography, the new norms can add to a more complete representation of Indian children. Regardless, the norm-referenced scores we have provided are likely closer to that of the total child population than the original norm-referenced scores, as the majority of Indian children attend government schools (Government of India, 2021–22; Narwana et al., 2026).

We acknowledge that our sample, and therefore our analysis, has some limitations. Most notable, the participating children were from three different cities/regions: Delhi, Hyderabad, and Patna were selected to capture geographic, linguistic, and socio-cultural diversity across different regions of India (North and South, as well as a major metropolitan versus a less-resourced urban context). While this does not fully capture the heterogeneity of the Indian child population, it reflects a deliberate effort to represent children from across India. At the same time, the projects had to be pragmatic and balance the aim of representativeness with the logistical and feasibility constraints inherent to large-scale data collection. We acknowledge the complexities of sampling across a country as large and diverse as India, but follow the guidance proposed by Timmerman et al. (2025) who suggest that where it is unfeasible to include a fully representative sample, “it can be a better choice to include standardized scores based on a small[er] sample” (p. 15). This may lead to limited generalizability, however. In addition, our sample only spanned between the ages of 8 and 11, in contrast with the original Indian norms, which started from the age of 4. Moreover, we had a distinct focus on children attending government schools, which has the advantage of being able to provide norms for this specific sub-population, but limits generalizability to other sub-samples of children. Nevertheless, we were able to present alternative norms based on a large sample size and valid representation of a sub-sample of the population.

A second limitation of this research lies in the datasets. With respect to the demographic data, much information about children's backgrounds, parental occupations and parental education was not reported. In addition, in contrast with many studies based in Western countries, the information about the children's backgrounds was not based on parental reports, but on children’s self-reports. In some cases, this made the information unreliable or less accurate (e.g., some children did not know their parents' profession, and some children reported speaking English whereas we can infer that this was likely only reported because the child thought that this was a socially desirable response). No other data was available in the examined datasets, but future work might try to obtain parental reports as well, in addition to other variables that have been reported to influence Raven’s performance, such as videogame experience, cellphone use, and (social) media consumption (Flynn & Shayer, 2018). Note, however, that this may be challenging and labor-intensive in instances in which parents cannot read or write, or where children are not meta-cognitively aware of their time engagement with these activities. With respect to the Raven’s dataset, it did not contain item-level data, so analyses such as item difficulty or internal consistency could not be conducted (though note that these were not conducted in Raven, 2012 either; cf. Cotton et al., 2005). Finally, we reported the official medium of instruction of the schools that the children attended, but from previous research we know that many different languages are used in the classroom in government schools in India (Lightfoot et al., 2021).

### Conclusion

In conclusion, this study challenges the existing norms for the Raven’s CPM in India, illustrating their limited applicability to a large and diverse sample who are not comparably socio-economically privileged as the sample on which the current norms are based. We provide alternative norms, which better reflect government school children’s educational and socio-demographic backgrounds and are more suitable to use with similar children than existing norms. It is not necessary to replace the existing Raven’s CPM norms completely, but these new norms can be used by researchers, educators and clinicians where appropriate.

## Data Availability

The datasets analyzed during the current study are available on the Open Science Framework repository, along with all analysis code: https://osf.io/4tm3u/overview?view_only=434e59262a654fc4b25d2c144d0da179.

## Footnotes

1 Also see https://www.pib.gov.in/PressReleasePage.aspx?PRID=2097864&reg=3&lang=2#:~:text=Government%20schools%20comprises%2069%20%25%20of,Ratio%20(GER)%20by%202030. Accessed 08 May 2026.

2 Such English idealism in low-cost private as well as government school contexts does not always translate to ‘pure’ English use in practice though (Lightfoot et al., 2022; Treffers-Daller et al., 2022; Tsimpli et al., 2020a; 2020b; Tsimpli & Balasubramanian, 2024). Nevertheless, the goal of private education is to implement English instruction, often without taking learners' diverse linguistic backgrounds and English exposure into account.

3 There may be concern about the temporal gap between the two datasets and the potential influence of COVID-19 on children’s schooling and cognitive performance. Accordingly, we compared the ASCI dataset to age-matched data from Hyderabad from the MultiLiLa project. The children in the ASCI dataset scored higher (mean raw score: 23.7) than their MultiLiLa peers (mean raw score: 20.5; unpaired t-test: *t* = 5.47; df = 409; *p* < .001) on the CPM, not lower as might be expected after COVID-19. This performance difference may thus simply reflect naturally varying performance levels across regions, neighborhoods, schools, and individual children. Further investigation into this effect is beyond the scope of the current study.

4 Where it was not possible for the Research Assistant to speak the language most comfortable to the child, they found a language in which both could understand each other and used that to communicate.
